# Type 2 MI a legitimate efficacy endpoint in cardiovascular trials? A critical appraisal

**DOI:** 10.3389/fcvm.2025.1564432

**Published:** 2025-06-19

**Authors:** F. H. van Bruggen, H. J. Luijendijk

**Affiliations:** Department of Primary and Long-Term Care, University of Groningen, University Medical Centre Groningen, Groningen, Netherlands

**Keywords:** type 2 MI, PCSK 9 inhibitors, GLP 1 analog, SLGT inhibitors, efficacy outcomes, safety outcomes

## Introduction

Myocardial infarction (MI) is a critical endpoint in cardiovascular clinical outcomes trials, representing a diverse entity with distinct subtypes. The classification introduced in 2007 includes five MI subtypes: type 1 (spontaneous atherosclerotic), type 2 (oxygen supply-demand mismatch), type 3 (cardiac death without biomarker elevation), type 4 (percutaneous intervention-related), and type 5 (surgery-related) (1). Type 2 MI is caused by an imbalance between myocardial oxygen supply and demand in the absence of acute atherothrombotic plaque disruption, but it often occurs in the presence of underlying atherosclerotic coronary artery disease. This cause distinguishes it from type 1 MI, which is typically caused by coronary artery disease and acute thrombosis. Type 2 MI carries a higher mortality risk than type 1 MI (2).

The prevalence and incidence of type 2 MI are increasing. Studies have reported its prevalence among emergency department patients with suspected MI to range from 26% to 58% (3). The reported incidence of type 2 MI varies between 7% and 35%, depending on heterogeneity in populations and diagnostic criteria (4). With an aging population and rising comorbidities, its incidence is expected to grow exponentially (5–7).

The growing incidence of type 2 MI underscores the need for effective preventive therapies. Several recent clinical outcomes trials testing cholesterol- and glucose-lowering drugs have reported the effects on type 2 MI (8–11). Type 2 MIs were found to substantially contribute to the primary endpoints in these trials. These results necessitate a careful evaluation of the suitability of type 2 MI as a primary efficacy endpoint in clinical trials, particularly for medications aimed at reducing atherosclerosis-related events. In this paper, we examine the incidence of type 2 MI in these trials, evaluate proposed pathophysiological mechanisms, and explore the implications of including type 2 MI in primary endpoints.

## Risk factors for type 2 MI

Type 2 MI shares many cardiovascular risk factors with type 1 MI (12). Atherosclerosis has been reported to be present in about 30%–50% of type 2 MI cases (13, 14) and affects the prognosis negatively (15). Also, many patients with type 2 MI may have hyperlipidemia (12) or hypertension (16). Tachyarrhythmia may also precipitate type 2 MI through increased myocardial oxygen demand (16). Nonetheless, type 2 MI also has non-cardiovascular risk factors including operative stress, sepsis, anaemia, and respiratory failure (12). Viral and bacterial infections are also associated with an increased risk of type 2 MI (17, 18). Moreover, it is more common in females, older adults, and those with multiple comorbidities (18). Given its complex risk profile, a plausible mechanism linking lipid- and glucose-lowering therapies to type 2 MI is essential for including it as an efficacy endpoint (19, 20). Such a mechanism would strengthen the validity of the reported effects on composite endpoints.

## PCSK9-inhibitors and type 2 MI

Recent trials involving PCSK9 inhibitors have included type 2 MI in their primary endpoints (Table 1). The ODYSSEY OUTCOMES trial compared alirocumab to placebo in post-acute coronary syndrome patients on high-intensity or maximum-tolerated statin therapy. The primary endpoint included coronary heart disease death, nonfatal MI, fatal and nonfatal ischemic stroke, and unstable angina requiring hospitalization (8). MI itself was a composite outcome that included the various types of MI. In ODYSSEY OUTCOMES, 287 of 1,692 MIs (17.0%) were type 2, among 1,955 (14.7%) primary endpoints (8, 21). Alirocumab reduced the risk of type 2 MI (HR 0.77; 95% CI 0.61–0.97) (21).

**Table 1:** Contribution of type 2 MI to primary outcomes in cardiovascular clinical trials.

Trial name	Participants	Compared treatments	Primary outcome^a^	Observed number of primary endpoints	Number of MI (% of primary endpoints)	Number of type 2 MI (% of primary endpoints)
EMPA-REG OUTCOME, NEJM 2015	7,020 patients with T2DM and established CV disease of atherosclerotic origin	Empagliflozin 10 mg/day, 25 mg/day, or placebo	CV death, non-fatal MI, or non-fatal stroke	772	421 (54.5)	86 (11.1)
FOURIER, NEJM 2017	27,564 patients with ASCVD and LDL-C ≥ 70 mg/dl who were receiving statin therapy	Evolocumab vs. placebo (background statin therapy)	CV death, MI, stroke, hospitalization for unstable angina, or coronary revascularization	1,829^a^	1,107 (60.5)	176 (9.6)
ODYSSEY OUTCOMES, NEJM 2018	18,924 patients who had ACS 1–12 months earlier	Alirocumab vs. placebo (background statin at high-intensity or maximum tolerated dose)	Death from coronary heart disease, nonfatal MI, (non)fatal ischemic stroke, or unstable angina requiring hospitalization	1,955	1,692 (86.5)	287 (14.7)
Harmony Outcomes, Lancet 2018	9,463 patients >40 years of age with T2DM and established ASCVD	Albiglutide (30–50 mg) vs. placebo	CV death, MI, or stroke.	766	421 (54.9)	54 (7.0)

ACS, acute coronary syndrome; ASCVD, atherosclerotic cardiovascular disease; CV, cardiovascular; LDL-C, low-density lipoprotein cholesterol; MI, myocardial infarction; NEJM, New England Journal of Medicine; T2DM, type 2 diabetes mellitus.

^a^ As FOURIER was powered on the key secondary outcome, we reported the number of these events and the number of MI and type 2 MI related to the number of key secondary outcomes.

The FOURIER trial evaluated evolocumab vs. placebo in patients with prior MI or stroke on statin therapy (9). The primary composite endpoint included cardiovascular death, MI, stroke, hospitalization for unstable angina, and coronary revascularization. The trial was powered on the secondary endpoint of cardiovascular death, MI, or stroke. Again, MI was a composite outcome that included type 2 MI. FOURIER reported 176 type 2 MI out of 1,107 MIs (15.9%) among 1,829 secondary endpoints (9.6%) (9, 22),. Evolocumab showed a non-significant increase in type 2 MI risk (HR 1.09; 95% CI 0.82–1.44) (22). Thus, the effects of alirocumab and evolocumab on type 2 MI were inconsistent.

## LDL-C lowering and type 2 MI

The ODYSSEY OUTCOMES investigators attributed alirocumab's reduced type 2 MI risk to improved myocardial oxygen supply by preventing plaque progression or promoting regression (21). However, FOURIER's evolocumab group achieved a lower mean LDL-C (30 mg/dl or 0.8 mmol/L) than ODYSSEY OUTCOMES' alirocumab group (58 mg/dl or 1.5 mmol/L) (8, 9). Hence, a greater plaque regression would be expected with evolocumab, but this did not correlate with a reduced type 2 MI risk. Also, a multivariable Cox regression analysis did not show an association between baseline LDL-C and the risk of type 2 MI in ODYSSEY OUTCOMES (21). Notably, to our knowledge, this is the only study to date specifically investigating the association between baseline LDL-C levels and incident type 2 MI.

The ODYSSEY OUTCOMES investigators suggested that the observed differences in effects between the trials could stem from differences in patient populations, event numbers, follow-up duration, definitions, and adjudication processes (21). However, in our view FOURIER and ODYSSEY OUTCOMES shared many similarities in these respects: both trials investigated LDL-C lowering drugs in populations with a high cardiovascular risk, employed the Third Universal Definition of Myocardial Infarction for classifying MI events, and utilized local blinded clinical events committees for the adjudication of events. Also, the proportion of type 2 MI among total MIs was very close: 15.9% in FOURIER and 17.0% in ODYSSEY OUTCOMES.

The IMPROVE-IT trial that evaluated ezetimibe against placebo could have provided valuable insights about the effects of LDL-C lowering and the risk of type 2 MI as well (23). As the third major clinical outcomes trial on intensive lipid-lowering therapy, it randomized patients at the time when the classification of MI subtypes, including type 2 MI, was already introduced (24). Unfortunately, no distinction between subtypes of MI was made in this trial. So, the question remains what explains the discrepancy in the effect of evolocumab and alirocumab on risk of type 2 MI.

Another potential explanation for the discrepancy in the risk of type 2 MI between the PCSK9 inhibitors may lie in the observed differences in the risk of severe infection. A systematic review and meta-analysis examined the association between PCSK9 inhibitor use and infection risk (25). In FOURIER, the evolocumab group showed an increased risk of both severe viral (HR 1.26; 95% CI 0.81–1.96) and bacterial infections (HR 1.06; 95% CI 0.83–1.35) compared to placebo but power was insufficient to be certain. ODYSSEY OUTCOMES similarly showed an increased but not statistically significant risk of severe viral infections (HR 1.10; 95% CI 0.72–1.61) for alirocumab, but a lower risk of severe bacterial infections (HR 0.81; 95% CI 0.62–1.08) (25).

## Glucose-lowering and type 2 MI

Two clinical outcomes trials evaluating glucose-lowering drugs have also reported type 2 MI results. EMPA-REG OUTCOME evaluated the cardiovascular outcomes of empagliflozin 10 or 25 mg added to the participants' existing treatment regimen, compared to placebo. The study included patients with type 2 diabetes at high cardiovascular risk. The primary endpoint was a composite of death from cardiovascular causes, nonfatal myocardial infarction, or nonfatal stroke (11). In the end, 86 of 421 MIs (20.4%) were classified as type 2 MIs, among 772 (11.1%) primary endpoints (11, 26). Empagliflozin showed an adjusted rate ratio of 0.67 (95% CI, 0.41–1.10) for type 2 MI compared to placebo (26).

The HARMONY OUTCOMES trial assessed the cardiovascular efficacy of albiglutide in patients with type 2 diabetes at high cardiovascular risk. Participants were randomized to receive albiglutide (30–50 mg) or placebo, against a background of cardiovascular medication. The primary endpoint was a composite of cardiovascular death, MI, or stroke. In total, 54 of 421 MIs (12.8%) were type 2 MIs, among 766 (7.0%) primary endpoints (10, 27). Albiglutide exhibited a hazard ratio of 0.65 (95% CI, 0.46–0.92) for type 2 MI (27).

Both empagliflozin and albiglutide demonstrated a reduction in the risk of type 2 MI. The beneficial effect of empagliflozin, an SGLT2-inhibitor, was attributed to an improved cardiac oxygen supply-demand balance through multiple mechanisms, including increased hemoglobin levels, shifted cardiac metabolism, reduced plasma volume, and decreased myocardial oxygen demand (11). For albiglutide, an GLP-1 agonist, no explanatory mechanism was provided by the investigators. Nevertheless, other researchers have proposed that the cardiovascular benefits of GLP-1 agonists may arise from multiple mechanisms. These include direct cardioprotection, vasodilation, natriuresis, and anti-inflammatory effects (28), which could play a role in reducing the risk of type 2 MI. Further evidence is needed to substantiate the observed beneficial effects on type 2 MI. Several other cardiovascular outcome trials investigating glucose-lowering drugs also included type 2 MI as part of the primary outcomes, but the results regarding type 2 MI have not been published yet (29–32).

## Conclusion

As the prevalence and incidence of type 2 MI is growing, there is a pressing need for more evidence about the impact of cardiovascular drug therapy in the management and prevention of type 2 MI in the absence of atherosclerosis (33). Including type 2 MI in the primary endpoints of cardiovascular outcome trials requires careful consideration to ensure the validity and interpretability of study results. The appropriateness of this approach may vary depending on the intervention being studied and the available evidence supporting its effects on type 2 MI risk. A crucial first step is establishing a plausible pathophysiological mechanism between the intervention and the risk of type 2 MI (19, 20). Future clinical trials should explicitly report the impact of the investigated therapy on the risk of type 2 MI. Additionally, clinical trials specifically designed for populations at high risk of type 2 MI are needed to provide deeper insights into the effects of medications aimed at preventing atherosclerosis. For the time being, researchers should exercise caution when considering type 2 MI as an efficacy endpoint in trials about preventive cardiovascular drugs. It may be more appropriate to focus on well-established atherosclerosis-related endpoints for assessing efficacy of drugs aimed at reducing atherosclerosis, while monitoring type 2 MI as a safety outcome.
